# Contribution of Various Types of Transfusion to Acute and Delayed Intracerebral Hemorrhage Injury

**DOI:** 10.3389/fneur.2021.727569

**Published:** 2021-10-29

**Authors:** Siddharth Kumar, Matthew Andoniadis, Ali Solhpour, Salman Asghar, Madison Fangman, Rani Ashouri, Sylvain Doré

**Affiliations:** ^1^Department of Anesthesiology, University of Florida College of Medicine, Gainesville, FL, United States; ^2^Departments of Psychiatry, Pharmaceutics, Psychology, and Neuroscience, Center for Translational Research in Neurodegenerative Disease, McKnight Brain Institute, University of Florida College of Medicine, Gainesville, FL, United States

**Keywords:** edema, acute and delayed injury, intracerebral hemorrhage, transfusion, secondary injury, hematoma expansion

## Abstract

Intracerebral hemorrhage (ICH) is the second most prevalent type of stroke, after ischemic stroke, and has exceptionally high morbidity and mortality rates. After spontaneous ICH, one primary goal is to restrict hematoma expansion, and the second is to limit brain edema and secondary injury. Various types of transfusion therapies have been studied as treatment options to alleviate the adverse effects of ICH etiopathology. The objective of this work is to review transfusions with platelets, fresh frozen plasma (FFP), prothrombin complex concentrate (PCC), and red blood cells (RBCs) in patients with ICH. Furthermore, tranexamic acid infusion studies have been included due to its connection to ICH and hematoma expansion. As stated, the first line of therapy is limiting bleeding in the brain and hematoma expansion. Platelet transfusion is used to promote recovery and mitigate brain damage, notably in patients with severe thrombocytopenia. Additionally, tranexamic acid infusion, FFP, and PCC transfusion have been shown to affect hematoma expansion rate and volume. Although there is limited available research, RBC transfusions have been shown to cause higher tissue oxygenation and lower mortality, notably after brain edema, increases in intracranial pressure, and hypoxia. However, these types of transfusion have varied results depending on the patient, hemostasis status/blood thinner, hemolysis, anemia, and complications, among other variables. Inconsistencies in published results on various transfusion therapies led us to review the data and discuss issues that need to be considered when establishing future guidelines for patients with ICH.

## Introduction

Intracerebral hemorrhage (ICH) is the second most prevalent type of stroke, behind ischemic stroke, and has exceptionally high morbidity and mortality rates, with 5.3 million cases and 3 million reported deaths worldwide in 2010. Case fatality rates reach nearly 60% at 1 year after stroke, and only 20% of patients who survive become independent within 6 months of injury (1). ICH is the most common form of hemorrhagic stroke, resulting from bleeding in the brain tissue and ventricles and caused by hypertension, arteriovenous malformations, or head trauma. Pathophysiological considerations of ICH involve inflammation, edema, iron toxicity, oxidative stress, and thrombin formation. The primary injury leading to compression of the brain is the development of the hematoma, a collection of blood outside of blood vessels. Accordingly, the hematoma would increase the intracranial pressure (ICP), leading to brain hernias caused by a lack of blood flow (2).

### Pathophysiology

ICH hematoma expansion is a frequent phenomenon that occurs in 70% of cases, and the majority (26%) of relevant growth occurs within 4 h of symptom onset; therefore, a repeat early computed tomography (CT) scan is needed to detect it (3). Hematoma expansion can be prevented, and urgent identification of patients with a high risk of active bleeding is crucial (4). In patients with ICH, intraventricular hemorrhage imaging is frequently present upon admission and is associated with poor long-term outcomes. Delayed intraventricular hemorrhage on subsequent scans is far less common and appears to be associated with a better outcome (5). The pathophysiology of early hematoma expansion remains unclear; however, it could result from leakage, rebleeding, or structural damage to the immediate surrounding environment. The primary reason is supportive and protective tissue disruption, but other factors such as elevated ICP and reduced oxygen supply might act on vessels and the blood-brain barrier (BBB) (3). According to Naidech et al. and others, coagulopathies or anticoagulants can also account for repeated or continuous bleeding, disturbing autoregulation (6–8). In addition, hypertension initiates uncontrolled perfusion pressure, which causes further bleeding. Death and physiological dysfunction will often result from the secondary expansion of hematoma after spontaneous intracerebral hemorrhage. Hematoma expansion includes all forms of the extended three-dimensional distribution of the initial hemorrhage, including intraparenchymal or intraventricular volume enlargement or transition, invasion, or rebleeding into compartments, to the side of the original bleeding but excluding perihematomal edema (9). Furthermore, a hematoma expansion is considered early when it occurs within the first 24 h of ICH onset (3). A late hematoma expansion occurs from days 2–14 and days 14–28 (10).

ICH and other patients in the neuro-intensive care unit (ICU) require treatment that is different than that for most regular ICU patients. They often require more invasive hemodynamic and intracranial monitoring systems along with tracheostomy but with fewer intravenous sedations than regular ICU patients (11). The surgical evacuation of the hematoma can be performed via stereotactic aspiration, endoscopic surgery and craniotomy, and the comparison of safety and efficacy of these methods have been investigated before (12). Furthermore, to help with the increase in ICP and brain swelling, decompressive craniectomy may be indicated, but it also has many caveats (13).

### Risk Factors for Hematoma Expansion

Naidech et al. in 2009 investigated patient and treatment characteristics such as hematoma volume, intraventricular invasion, early neurological deterioration, treatment with recombinant coagulation factor VIIa, and blood pressure treatment. Ederies et al. in 2009 studied radiological characteristics such as shorter time between onset and first CT, hematoma density heterogeneity on admission CT, and occurrence of a “spot sign” in CT angiography. Delgado et al. studied laboratory characteristics such as reduced platelet activity and elevated IL-6, cellular fibronectin, and D-dimers (14).

Three groups and their respective studies have described prior use of platelets (6, 15, 16). The authors described platelets as a risk factor for hematoma growth in patients with ICH treated with tranexamic acid and antihypertensives within 24 h; however, one limitation in this study is the uncertainty in the timing of CT scans performed. Goldstein is credited with contrast extravasation on admission CT angiography (“spot sign”) as a predictor of hematoma expansion, leading to Delgado et al. proposing a “spot sign score” in 2006. Independent predictors of poor outcomes were presenting hematoma volume, expansion of hematoma volume, and the development of intraventricular hemorrhage, shown in the landmark studies by the Davis and Tuhrim groups (17, 18). A landmark paper by Ohwaki et al. proved conclusively that maximum systolic blood pressure of 150 mmHg was independently associated with hematoma expansion in 76 patients with spontaneous ICH (19). Another large trial addressed the effect of lowering blood pressure in ICH (20). The INTEnsive blood pressure Reduction in Acute Cerebral hemorrhage Trial (INTERACT) studied rapid blood pressure reduction within the first 6 h, which was shown to be safe and feasible and to reduce hematoma expansion. A limitation of the study was that there was no difference in outcome when compared with similar studies. Another prospective trial investigating this issue is the Antihypertensive Treatment of Acute Cerebral Hemorrhage (ATACH) trial. The authors reported in this Phase I dose-finding trial that the treatment was safe and feasible (21).

Brain edema and herniation can cause secondary injuries, leading to increased mortality rates and poorer outcomes in patients with ICH (2). ICH can also result from severe complications of oral anticoagulant therapy, with mortality levels reaching over 50%. The medications used to prevent blood clottings, such as vitamin K antagonists and newer oral anticoagulant drugs, increase the likelihood of ICH and account for more than 15% of all cases (1). ICH is life-threatening due to continuous bleeding, which can cause massive pressure buildup in the brain, leading to a midline shift of the brain and compressing vital structures. Other etiological factors of ICH include arteriovenous malformation or hypertension. ICH has been thoroughly reviewed in research, as seen in Table 1, with numerous proposed treatment plans that have had a limited impact on recovery. Over the years, treatment options for ICH have examined platelet transfusion and red blood cell (RBC) transfusion. Platelet transfusion allows for platelet activation and, thus, potentially reduces hemorrhage size (22). In contrast, RBC transfusion restores hemoglobin (Hgb) levels and can ameliorate anemia in these patients, although additional research is needed to elucidate its effectiveness fully. Various transfusion treatment options may be available to limit hematoma expansion, as well as delayed brain injury associated with ICH. Overall, current data are limited and ambiguous regarding the relationship between transfusion therapies and their benefit in treating patients with ICH. However, what is known is that it is essential to first mitigate the hematoma size and expansion rate and then limit brain edema and secondary injury. This review discusses the current state of research, the purpose of transfusion therapy, problems related to this course of treatment, and putative considerations for future research in this field.

**Table 1:** Summary of studies that have used blood product transfusion and tranexamic acid infusion therapy in patients with ICH.

**Transfusion/Infusion Type**	**References**	**Study type/Years of study/Sample size (Sex %)/Age**	**Variables**	**Transfusion measurements**	**Transfusion amount added or end goal**	**Clinical endpoints**	**Outcomes**
Platelet	Guerrero et al. (23) Variability in the use of platelet transfusion in patients with intracerebral hemorrhage: observations from the ethnic/racial variations of intracerebral hemorrhage study.	• Prospective • Date Range N/A • Total Sample: 2,572 • (60.0% M) • Platelet Transfused: 302 • (60.3% M) • Transfused Mean Age: 64.2 yr • Non-transfused Mean Age: 60.4 yr	Platelet count, external ventricular drain, hematoma evacuation, intracranial pressure monitoring	Platelet count for transfused: <50,000 8, 51,000–1,000:39, 101,000–150,000:32, 150,000–200,000: 545, >200,000: 161Not transfused: <50,000:10, 51,000–100,000:33, 101,000–150,000: 222, 150,000–200,000: 545, >200,000: 1,409	N/D	ICH, mortality, worse outcomes (mRS>4), volume of hematoma	Platelet therapy was associated with antiplatelet use before onset (OR: 5.02, 95% CI: 3.81 to 6.61, *p* <0.0001), thrombocytopenia (OR: 13.53, 95% CI: 8.43–21.72, *p* <0.0001), and ventriculostomy placement (OR: 1.85, 95% CI: 1.36–2.52, *p* <0.0001). At 3 mo, there was no difference in mortality or poor outcome in platelet therapy groups and groups that did not receive platelets.
Platelet	Baschin et al. (24) Platelet transfusion to reverse antiplatelet therapy before decompressive surgery in patients with intracranial hemorrhage.	• Retrospective • 2012–2014 • Total Sample: 72 • (69.4% M) • Received acetylsalicylic acid and P2Y12 inhibitor: 14 • Received acetylsalicylic acid: 53 • Received clopidogrel: 5 • Median Age: 75 yr	Sex, age, prior anticoagulation, antiplatelet therapy, cause of bleeding, onset of bleeding, location of bleeding, surgical procedure	N/D	19 patients received at least 2 units of RBC concentrates	Frequency of new arterial thrombotic complications and frequency of recurrent ICH	Low risk for cardio-cerebral thrombotic complications was shown by platelet transfusion before cranial decompressive surgery in patients with ICH complicating antiplatelet therapy. Patients with chronic ICH and patients treated with clopidogrel had a higher risk of rebleeding.
Platelet	Ducruet et al. (25) Impact of platelet transfusion on hematoma expansion in patients receiving antiplatelet agents before intracerebral hemorrhage.	• Retrospective • 06-2003-07-2008 • Total Sample: 66 • Platelet Transfused: 35 (58.9% M) • Non-transfused: 31 • (58.1% M) • Platelet Group Age: 73.2 ± 10.1 yr • No platelet Group Age: 71.7 ± 13.5 yr	Clopidogrel, hypertension, GCS score, myocardial infarction, deep venous thrombosis, cerebrovascular accident, kidney failure, pneumonia, urinary tract infection, bacteremia, initial hematoma volume, final hematoma volume, absolute change in volume, days in NICU, days in hospital, mortality	N/D	N/D	Rate of significant hematoma expansion in transfused vs. non-transfused patients. Discharged mRS and rates of systemic complications	The study suggests that platelet administration does not reduce the frequency of hematoma expansion in patients with ICH receiving antiplatelet therapy. Transfusion timing may play a role in this because hematoma expansion typically occurs during the first 6 h after a stroke.
Platelet	Chen et al. (26) Effects of platelet infusion, anticoagulant, and other risk factors on the rehemorrhagia after surgery of hypertensive cerebral hemorrhage.	• Selective trial • 04-2007-06-2012 • Total sample: 269 • (58% M) • Platelet Received: 186 • Age: 67.1 ± 12.1 yr	GCS score at admission, symptoms including headache, nausea, vomiting, complications including hemiplegia, meningeal intracranial infection, multiple organ failure, acute renal failure, urinary	N/D	N/D	Re-hemorrhage after surgery for HCH	A decreased risk of hemorrhage recurrence was associated with platelet transfusion. Out of 186 patients who received platelet infusions, 18 had hemorrhage recurrences, whereas 16 had recurrences among the other 83 patients who did not receive platelet infusions. Analysis indicated a significant difference between these two groups (*p* <0.05).
			system infection, dysphoria, Glasgow outcome scale score, and coagulation function data				
Platelet	Naidech et al. (22) Early platelet transfusion improves platelet activity and may improve outcomes after intracerebral hemorrhage.	• Prospective • Date Range: N/A • Total Sample: 45 • (58% M) • Age: 67.3 ± 14.0	ICH score, age, aspirin use, clopidogrel use, intraventricular hemorrhage, craniotomy, blood pressure, initial and follow-up hematoma size, mRS <4 at 3 mo, minutes from symptom onset to diagnostic CT	N/D	N/D	Hemorrhage size, platelet activity, patient outcome	Platelet transfusion was shown to significantly change platelet activity assays in ICH patients. In patients identified as being at high risk for poor outcome and hemorrhage growth, administration of platelets within 6 or 12 h of symptom onset was associated with a smaller final hemorrhage size as well as improved odds of independence at 3 months.
FFP & PCC	Steiner et al. (27) Fresh frozen plasma vs. prothrombin complex concentrate in patients with intracranial hemorrhage related to vitamin K antagonists (INCH): a randomized trial.	• Randomized trial • 08-2009-01-2015 • Total sample: 54 • (62% M) • Mean Age:75.6 yr	Sex, INR, mean systolic, and diastolic blood pressure, median hematoma volume, mean body mass index, diabetes, hypertension, mRS, GCS score	N/D	N/D	INR 1–2 or lower, anticoagulation reversal, death, hematoma expansion by day 90 after treatment, mRS, NIHSS, quality of life at day 90	When studying FFP vs. PCC, patients with ICH related to vitamin K antagonists (VKA-ICH) showed more promising results regarding hematoma expansion rates when using PCC. Eight of the FFP patients died 48 h after symptom onset, 5 from hematoma expansion. PCC patients only had 5 deaths, but none were related to hematoma expansion.
FFP & PCC	Huttner et al. (28) Hematoma growth and outcome in treated neurocritical care patients with intracerebral hemorrhage related to oral anticoagulant therapy: comparison of acute treatment strategies using vitamin K, fresh frozen plasma, and prothrombin complex concentrates	• Retrospective • 01-1999-12-2003 • Total sample: 55 • PCC alone or in combination with FFP and VAK: 31 (58% M) • FFP alone or in combination with VAK: 18 • (61% M) • VAK only: 6 • (66% M) • PCC Group Mean Age: 68 yr • FFP Group Mean Age: 70 yr • VAK only Mean Age: 71 yr	Gender, age, GCS score, glucose, cholesterol, mean arterial pressure, treatment regimens	N/D	N/D	Hematoma volume, INR, hematoma expansion	Oral anticoagulant therapy patients with ICH who had undergone PCC transfusion had a more significant reduction in the hematoma expansion rate than patients who received FFP transfusion. The INR reversal was quicker in PCC patients than in FFP patients.
PRBC	Roh et al. (29) Red blood cell transfusions and outcomes after intracerebral hemorrhage.	• Retrospective • 2002-2011 • Total sample: 587,046 • PRBC transfused: 3.8% (47.3% M) • No PRBC transfused: 96.2% • (47.5% M) • Age range: • 65–79 yr	In-hospital mortality, home discharge, unfavorable discharge, favorable discharge, modified Charlson Comorbidity Index, cases with anemia	N/D	N/D	ICH, Charlson Comorbidity Index, comorbidities, hospitalization length	The effects of PRBC transfusion on patients with ICH were mixed. When looking at patients requiring mechanical ventilation, transfusion was related to poor outcomes (OR: 1.33, 95% CI: 1.27–1.39). However, there was no significant association in cases without mechanical ventilation (OR: 0.88, 95% CI: 0.83–1.13). In addition, patients with ICH requiring external ventriculostomy and PRBC treatment (16.1%) did not have poor ICH outcomes (OR: 1.05, 95% CI: 0.97–1.10), whereas patients not
							requiring ventriculostomy had poor ICH outcomes (OR: 1.51, 95% CI: 1.46–1.57).
PRBC	Ibrahim (30) Blood transfusion does not improve outcomes in patients with spontaneous intracerebral hemorrhage.	• Retrospective • Date Range: N/A • Total Sample: 163 (51.5% M) • PRBC transfused: 25 • Median age: 59 yr	Mortality, Hgb level, morbidity, length of stay (LOS), poor outcome	Patients with Hgb <8 g/dL received a transfusion	N/D	ICH, length of stay, mortality, morbidity	Based on a univariate analysis, patients receiving PRBC therapy were shown to have significantly increased 30 d mortality. PRBC patients had a 30 d mortality of 48%, in contrast to patients without PRBC with 30 d mortality of 25.4% (*p* = 0.03). PRBC patients also had higher morbidity of 84 vs. 50.7% at *p* = 0.004. The length of stay in the hospital also increased to 11.7 ± 12.3 d from 7.5 ± 6.7 d (*p* = 0.01). However, multivariate logistical regression analysis did not show a significant effect caused by PRBC transfusion.
PRBC	Martin-Schild (31) Packed red blood cell transfusion is associated with adverse outcomes in ICH patients.	• Prospective • 2008–2010 • Total Sample: 111 • PRBC Transfused:19 • Age: N/D	Age, Hgb level, admission ICH score, intubation time, mRS score	N/D	N/D	ICH, NIHSS score, ICH score, mRS score	The study indicated that upon receiving PRBC transfusion, the NIHSS score was 17, compared to 15 for a patient not receiving PRBC (*p* = 0.021). The ICH score in PRBC patients was 2, compared to 1 in those not receiving PRBC (*p* = 0.013). Hgb levels were also lower at 9.3 vs. 14.0 g/dL in patients not undergoing transfusion (*p* = 0.001). Transfused patients also had a higher likelihood of needing intubation, at 84 vs. 39% (*p* = 0.0003). Transfused patients also had more significant poor functional outcomes, at 78.9 vs. 29.4%, with an mRS of 5–6 (*p* = 0.0002). Most importantly, transfused patients were 3 times more likely to die (OR: 2.99, 95% CI: 1.01–8.85, *p* = 0.047). However, when the data were adjusted for age, intubation, Hgb level, and admission ICH score, the data were not significant.
PRBC	Moman et al. (32) Red blood cell transfusion in acute brain injury subtypes: An observational cohort study.	• Retrospective • 2008–2015 • Total Sample: 2,638 • PRBC Transfused: 225 • (48% M) • Transfused median Age: 62 yr • Non-transfused Median Age: 68 yr	Use of RBC transfusion, age, gender, body mass index, comorbidities, Charlson Comorbidity Index score, GCS score, sequential organ failure assessment (SOFA) score albumin, creatinine, Hgb	8.0 g/dL	Between 7 and 10 g/dL	Length of stay, ICU length of stay, mortality, change in SOFA score 24 h after RBC transfusion	PRBC transfusion was related to increased hospital and ICU length of stay in patients with ICH with acute brain injury. However, mortality rates seemed to be lower. With these results, it is evident that further research is required. Using a restrictive transfusion protocol would be beneficial in allowing for PRBC transfusion to provide valuable results.
PRBC	Sheth et al. (33) Packed red blood cell transfusion and decreased mortality in intracerebral hemorrhage.	• Prospective and Retrospective • 1999–2005 • Total Sample: 546 • PRBC Transfused: 100 (55% M) • Transfused Median Age: 74 yr	Age, ICH volume, GCS score, location of hemorrhage	Mean baseline Hgb level: 13.0 g/dL	N/D	30 d mortality rate, ICH, GCS score, hemorrhage volume	The study indicates that anemia is a problem that can cause increased mortality in patients with ICH. Therefore, the use of PRBC transfusion may help mitigate the adverse effects of ICH. However, more research is necessary to determine this. A multivariate analysis controlled for admission GCS score, hematoma volume and location, anemia, warfarin use, and do not resuscitate status and found
		Non-transfused Median Age: 73 yr					that transfusion was related with a lower 30 d mortality (OR: 0.40, 95% CI: 0.19–0.69, *p* = 0.02).
PRBC	Chang et al. (34) Nadir hemoglobin is associated with poor outcome from intracerebral hemorrhage.	• Retrospective • 2008–2010 • Total Sample: 109 • Low Hgb: 30 (60.0% M) • Normal Hgb: 79 (51.9% M) • Low Hgb Median Age: 56.5 yr • Normal Hgb Median Age: 61.0 yr	Admit ICH score, systolic blood pressure, diastolic blood pressure, GCS score, nadir Hgb level	Low: 8.7 g/dLNormal: 12 g/dL	N/D	Length of stay, discharge NIHSS, discharge mRS	Patients who received PRBC transfusion, in an unadjusted model, showed that they had 9 times greater odds of having a discharge mRS of 5–6. However, the results were no longer significant when the data were adjusted for admission ICH score, nadir Hgb, age, and intubation, although they still showed a positive relation (OR: 4.01, 95% CI 0.64–25.32, *p* = 0.0473).
Tranexamic acid	Arumugam et al. (35) Tranexamic acid as antifibrinolytic agent in non-traumatic intracerebral hemorrhages.	• Randomized trial • 09-2012-10-2013 • Total Sample: 30 • (60% M) • Mean Age: • 52.93 yr	Age, sex, ethnicity, co-morbidities, hypertension, hypertension/diabetes mellitus, time of onset to first CT brain scan, time from first CT brain scan to treatment administration, admission GCS score, symptoms of presentation	N/D	N/D	Hematoma volume, hematoma expansion	The use of tranexamic acid was associated with the maintenance of hematoma volume. The treatment group's hematoma volume was initially measured at an average of 10.00 cm^3^ and the after-treatment value was 10.08 cm^3^ indicating no statistical difference in the size (*p* = 0.313). However, the control group had a significant hematoma volume growth of 3.07 cm^3^ (*p* = 0.001).
Tranexamic acid	Gao et al. (36) Tranexamic acid inhibits hematoma expansion in intracerebral hemorrhage and traumatic brain injury. Does blood pressure play a potential role? A meta-analysis from randomized controlled trials.	• Meta-analysis • 01-2001-05-2020 • Total sample: 1,553 • Age: N/D	Hematoma type, sample size, GCS score, treatment admission time, systolic blood pressure, ethnic origin, hematoma volume, hematoma expansion rate	N/D	N/D	GCS score, hematoma expansion rate, hematoma volume	In patients with ICH with moderate to severe hypertension (>160 mmHg), the tranexamic acid levels were associated with a lower rate of hematoma expansion (*p* = 0.02) and a decrease in hematoma volume (*p* = 0.04).
Tranexamic acid	Hu et al. (37) Tranexamic acid in cerebral hemorrhage: a meta-analysis and systematic review.	• Systematic review and meta-analysis • Studies until 09-2018 • Total Sample: 4,703 • Age: N/D	Sex, diagnosis, age, sample size, examination method	N/D	N/D	Mortality, GCS score >3, mRS>4, hydrocephalus, convulsions, seizures, hematoma expansion rate, hematoma volume	Patients with ICH undergoing tranexamic acid treatment showed improved 90-day mortality (OR: 0.99, 95% CI: 0.84–1.18; *p* = 95). The reduction in hematoma expansion (OR: 0.54, 95% CI: 0.37–0.80; *p* = 0.002) and volume (95% CI: −3.00 to −0.97, *p* = 0.0001) had a mean difference of −1.98 from the baseline value.

## Literature Search

Through extensive searching in four significant databases, PubMed, Google Scholar, OneSearch, and Dimensions, relevant articles from all possible years were searched with studies from 1982 to 2020 used to uncover the foundation of transfusion therapy research and build on our current understanding. We limited our search to articles written in English, as well as only those relating to humans. The following search terms were used in all databases: “blood transfusion and ICH,” “blood transfusion and intracerebral hemorrhage,” “transfusion and ICH,” “packed red blood cell (PRBC) and ICH,” “whole blood cell and ICH,” “platelet transfusion and ICH,” “tranexamic acid and ICH,” “PCC transfusion and ICH,” and “FFP transfusion and ICH.” We also expanded our search with different wording, for example, using the phrase “effects of PRBC transfusion on ICH patients.” Abbreviations were also incorporated if more articles could be found.

## Use of Blood Product Transfusion and Tranexamic Acid Infusion Therapies

The primary reason for using transfusion therapy in patients with ICH is to minimize hemorrhage volume and expansion. Additional reasons for using transfusion therapy include increasing blood perfusion and oxygen to promote adequate oxygen and nutrient delivery and slow hematoma expansion while mitigating delayed brain injuries. Due to sufficient blood loss, Hgb deficiency can cause improper oxygen delivery to muscles and tissues throughout the body. Using transfusion therapies makes it possible to restore Hgb levels and reestablish adequate oxygen delivery (38). Current indications for transfusion include blood replacement for surgical blood loss in certain circumstances with a significant Hgb level drop and ongoing bleeding or for patients with symptomatic anemia. Other indications include anemia, sickle cell disease (SCD), cancer, Hgb H disease, beta-thalassemia intermedia, beta-thalassemia major, and hemophilia, among other blood disorders (39). SCD, although not the focus of this paper, remains heavily reliant on transfusion therapy, but future SCD stroke treatments may focus on alternatives to blood transfusion therapy, such as hydroxyurea (40). There are multiple transfusion therapy types, including plasma transfusion, RBC transfusion, clotting factor transfusion, and platelet transfusion. Within these transfusion modalities, RBC and platelet transfusion are the most commonly used (41). Additionally, prothrombin complex concentrate (PCC) transfusion and tranexamic acid infusion have been studied. Tranexamic acid is a lysine-derived clotting promoter that has been shown to mitigate hematoma size and expansion compared to control groups (36). RBC transfusion can be further subdivided and categorized as either packed RBC (PRBC) transfusion, which contains only erythrocytes without the surrounding plasma content, or whole blood transfusion. RBC transfusion may help improve oxygen transport and delivery and restore original blood volume after ICH. On the other hand, in certain patients platelet therapy is used to stop bleeding during a hemorrhage to prevent further hematoma expansion. Platelets may help certain patients with ICH achieve better outcomes, as mentioned in the discussion section. For the prevention and treatment of bleeding in general medical cases, FFP, platelets, and cryoprecipitate are used (42). RBC transfusions are intended to improve tissue oxygenation in cases of anemia or acute blood loss due to trauma or surgery.

More than 90% of critically ill patients are anemic by the third day in the ICU (43). The multitude of factors implicated in anemia in critical illness includes decreased production of erythropoietin (EPO), inadequate EPO-induced bone marrow response, and diminished red cell survival, as well as treatment-associated traumatic blood loss (44). Almost half of all patients admitted to an ICU receive a blood transfusion, even though blood transfusions have been shown not to improve the outcome of patients in the ICU (44). Most patients with ICH are hospitalized in the ICU, many of whom have concomitant diseases. Thus, anemia may be common among this patient population, and they may be considered for blood transfusion.

The standard oxygen delivery equation (DO_2_) shows increasing oxygen delivery efficiency when increasing Hgb concentration. Blood transfusion increases oxygen use mostly in patients with Hgb below 4 g/dL (45, 46). Marik, Conrad, and Creteur and their colleagues studied oxygen consumption in critically ill patients, measuring it before and up to 6 h after a blood transfusion, but they failed to conclusively prove an increase in oxygen use and tissue oxygen tension after such transfusions (47–49). There are other significant but less-recognized risks of RBC transfusion related to the effects of blood storage and the immunomodulating effects of such transfusions, which occur in almost all recipients (50, 51). Non-infectious complications, which were shown in the 2009 study by Marik, should also be considered (52). A study conducted in healthy volunteers reported the presence of higher extravascular hemolysis after older RBC transfusion (storage of 40–42 days) than fresh blood (storage of 3–7 days) (53). As for when to start transfusion, patients who required transfusion have been administered it at certain trigger values throughout the years. The Canadian Critical Care Trials Group Study (TRICC) randomized 838 adult patients in the ICU to a transfusion trigger Hgb value of <7 or 10 g/dL (54). The TRIPICU study randomized 889 pediatric patients in the ICU to a transfusion trigger Hgb of <7 or <9.5 g/dL (55). Villanueva and colleagues randomized 921 patients with severe acute upper gastrointestinal bleeding to a transfusion trigger Hgb value of <7 or <9 g/dL (56). These studies indicate that the common transfusion trigger Hgb value is between 7 and 10 g/dL.

The Scientific Subcommittee on Disseminated Intravascular Coagulation (DIC) of the International Society on Thrombosis and Haemostasis (ISTH) has suggested that DIC be considered an acquired syndrome that is represented as an intravascular coagulation activation with a loss of localization originating from different causes. This is when blood clots form inside the blood vessels, using up much of the available clotting factors and leading to bleeding in other areas. This can cause damage to the microvasculature of the brain. If it is severe enough, it can lead to organ dysfunction (57).

## Issues Surrounding Transfusion Therapy

Although transfusion therapy produces rapid results, adverse effects might be a concern (58). It is essential to ensure blood or platelet therapy compatibility to limit side effects and promote proper acquisition. Another important consideration is that RBC storage affects the physical characteristics of RBCs. Changes in surface-to-volume ratio, cell shape, and osmotic rigidity are among the reported adverse effects of stored RBCs. These morphological changes are a limitation of using transfusion therapies because they have been associated with lower effectiveness in patients and adverse or worsened outcomes. One example of the detrimental effects of storing blood is the decrease in the post-transfusion 24-h RBC survival rate due to the change in the cell's surface-to-volume ratio. These unfavorable alterations are associated with impaired oxygen delivery and ATP depletion, lowering microvascular perfusion (38). Furthermore, stored cells release cytokines that can have an undesirable effect on the patient and may be associated with numerous adverse effects such as changing the sensitivity and expression of IL-6, IL-8, T cells, and TNFα (58). Future in-depth studies on the storage and time point of the delivery of blood and its effects, specifically on patients with ICH, remain an important consideration.

The lack of response from transfused blood to correct tissue perfusion might be due to biochemical and biomechanical changes termed the storage lesion. Decreased oxygen delivery to tissues results from storage lesions. After storage for 7 days, blood is depleted of 2,3-diphosphoglycerate, a compound that increases oxygen release from Hgb to tissues (59, 60). This shifts the oxygen dissociation curve to the left, reducing the available oxygen for tissue consumption. Increased storage time leads to acidemia and hyperkalemia, culminating in RBC lysis and release of free Hgb. Free Hgb scavenges nitric oxide and, therefore, may result in vasoconstriction and exacerbation of organ dysfunction (61–63). Structural changes due to RBC storage compromise microvascular circulation (64). The deformation of the biconcave structure of the 8-μm erythrocyte makes it difficult to navigate smaller capillaries and may result in vessel occlusion. The microvesiculation and loss of surface-to-volume ratio result in sphero-echinocytes. The formation of microvesicles denotes high osmotic fragility and diminished RBC survival (65, 66). Corpuscular changes occur because of ATP depletion (67). Vasoconstriction due to lysophosphatidylcholine species released from the cellular membrane of senescent RBCs is the cause of storage longer than 42 days (68). Increased RBC aggregability and adhesion compromises microvascular circulation (69, 70). Leukocyte changes due to the storage lesion also result in clinical side effects and transmitted infections due to contaminated leukocytes (71).

### Effect on Intracerebral Hemorrhage

Kumar et al. demonstrated a dose-dependent relationship between anemia and ICH volume (72). Sheth et al. reported that RBC transfusion was associated with improved survival at 30 days (OR: 2.76, 95% CI: 1.45–5.26, *p* = 0.002) and decreased mortality at 30 days (OR: 0.40, 95% CI: 0.19–0.69, *p* = 0.02). Despite transfusion, there was no significant increase in Hgb concentration (33). Hence, the protective mechanism remains unknown. Whether increased Hgb is required in the post-ICH period requires more research.

## Discussion

### Platelet Transfusion

A platelet count below 175,000/μL has been noted to be a significant predictor of ICH progression. Furthermore, patients with ICH with a platelet count below 100,000/μL have been associated with a 9 times greater risk of death (OR: 9.5, 95% CI: 1.3–71.4, adjusted *p* = 0.029) (73). There is significantly more research on platelet transfusion in patients with ICH than on RBC transfusions in acute and delayed ICH injuries, although published data reveal conflicting results. One study includes results that show patients who were considered high risk for hemorrhage growth had a decrease in modified Rankin Scale (mRS) scores upon early platelet transfusion (22). Groups that received platelet treatment <12 h after symptom onset had a better mRS (score <4) than groups who received it after 12 h, 3 months after treatment (11/20 patients compared to 0/7 patients, *p* = 0.01). However, this study has limitations because the patients were not randomly chosen for treatment and the sample size was too small for multivariate analysis.

The use of platelet transfusion has also been shown to be beneficial in patients undergoing tissue plasminogen activator (tPA) therapy. The tPA thrombolysis induces an increased risk in ICH formation, which is fatal in patients with ischemic stroke after tPA treatment (74). One study with mice highlighted that platelets could safeguard BBB integrity, suggesting that resting platelet transfusion can be a viable treatment option for improving tPA thrombolysis safety in ischemic stroke and patients with ICH (75). This report showed that platelet transfusion could significantly block the tPA-associated loss of cerebrovascular integrity and protect BBB permeability (75). Another study conducted by Baschin et al. reported that platelet concentrate transfusion given before cranial decompressive surgery in patients with ICH correlated with a low risk for cardio-cerebral thrombotic complications (24). Furthermore, when platelet transfusion coincided with surgical treatment in patients with ICH, it was administered with no reported issues, indicating that surgery can be performed safely after platelet transfusion (76). Additionally, clinical studies have shown that platelet transfusion can lead to a lower mRS score in patients with ICH. This finding indicates that platelet transfusion may be a viable ICH treatment option, although more support is needed in addition to a lower mRS score to solidify this claim (22).

A radiographic analysis by Ducruet et al. found no significant decrease in hematoma volume in either the ICH group treated with platelets or the ICH group without platelet treatment, with initial values of 27.7 ± 25.4 compared to 30.9 ± 28.3 (*p* = 0.63) and final values of 33.1 ± 30.8 compared to 33.9 ± 32.6 (*p* = 0.92) (25). However, after surgery for hypertensive cerebral hemorrhage, platelet use has been noted to prevent hemorrhage recurrence. A 2015 study by Chen et al. showed that after surgery, out of the 186 patients receiving platelet therapy, 18 patients had rebleeding, whereas 16 out of the 83 non-transfused patients rebled. The difference was shown to be statistically significant with a χ^2^-value of 4.790 (*p* = 0.045) (26). The use of platelet transfusion in patients with ICH undergoing antiplatelet therapy is an area of controversy. The American Association of Blood Banks' platelet guidelines in 2015 reported a lack of data on this topic and did not recommend transfusion in these particular cases (77). Another study showed that among patients with acute leukemia, the risk of ICH was higher among patients with low platelet counts and after receiving more platelet transfusions. However, the latter is potentially due to clinical factors leading to increased transfusion needs (78). On the other hand, prophylactic platelet transfusion for medical procedures has been associated with thrombosis and poor outcomes, including death. Most deaths were due to infection, sepsis, or organ failure, and none were due to bleeding or thrombosis (79).

It should be noted that in standard ICU settings, platelet use is reasonable for patients using antiplatelet agents (80). However, it is still important to evaluate the degree of hematoma growth because it is an independent indicator of mortality (17). This is because the mass effect of the primary bleeding can lead to an increase in ICP caused by the migration of lesions into the ventricles. Hematoma can also cause local edema and neurological damage in the parenchyma (81). Patients with a Glasgow Coma Scale (GCS) score of 8 or less and parenchymal hemorrhage volume ≥60 mL from their first CT have a predicted 30-day mortality rate of 91% (82). The perihematomal edema is promoted by thrombin within the hematoma. This can be dangerous because heme, iron, and Hgb can lead to cell death because they are strong mitochondrial toxins.

It has also been reported that patients on antiplatelet therapy with isolated ICH had worse mRS (OR: 3.6) after platelet transfusion than when first admitted to a treatment facility (83). Additionally, patients with isolated ICH had trauma-induced platelet dysfunction, whereas patients using aspirin had drug-induced abnormalities related to platelets in response to arachidonic acid. These results indicate that platelet transfusion does not improve trauma-induced platelet dysfunction but does improve aspirin-induced platelet dysfunction, further emphasizing the conflicting results published around transfusion therapy (84). However, ICH occurs in a closed space of the head; thus, the bleeding will cease in this space, resulting in the patient not losing a high volume of blood. Furthermore, a deficiency of platelet and coagulation factors is not significant enough on its own to instigate platelet transfusion. But the problems occur when patients have underlying diseases with platelet dysfunction or are using antiplatelet medications. In this scenario, the pressure effect of the expanded hematoma increases ICP due to the surrounding edema or hydrocephalus and may contribute to brain injury and neurologic deterioration. In this situation, the need for transfusion is highlighted for patients with severe thrombocytopenia and platelet dysfunction. Donor screening procedures and pathogen inactivation do not eliminate the risk of bacterial and other blood-borne infections, and infection by bacterially contaminated platelets represents a potentially serious problem with platelet transfusion (85). This is because platelets are stored at room temperature where bacteria can proliferate rapidly. The incidence of bacterial contamination is higher for platelets (~1 in 2,000) than it is for RBCs (~1 in 30,000) (86).

Overall, patients with severe thrombocytopenia may benefit from platelet transfusion. The threshold for transfusion varies case by case based on hematoma expansion, underlying disease, and history of antiplatelet or anticoagulant use. For patients on antiplatelet therapy, the available data suggest that platelet transfusions may be hazardous and should be avoided. However, further studies are warranted to compare different antiplatelet medications because some may lead to more bleeding than others. Moreover, it seems in a small number of patients that there is a likelihood for rebleeding and hematoma expansion in the first several hours of hemorrhage. This finding highlights the potential for platelet transfusion in the first hours of hemorrhage to lead to severe thrombocytopenia or platelet dysfunctions with expanding hematoma.

A 2017 study by Guerrero et al. showed that various factors such as prior use of antiplatelets (OR: 5.02, 95% CI: 3.81–6.61, *p* < 0.00001), thrombocytopenia (OR: 13.53, 95% CI: 8.43–21.72, *p* < 0.00001), and ventriculostomy placement (OR: 1.85, 95% CI: 1.36–2.52, *p* < 0.00001) were significantly associated with platelet therapy use in ICH patients. In addition, an interesting field of research might be the association of race and transfusion therapy in ICH patients, as the study showed that Black individuals were less likely to receive platelet transfusion therapy in this case (OR: 0.57, 95% CI: 0.41–0.80) (23). Furthermore, the ambiguity of the effectiveness of transfusion in patients with ICH is evident in anticoagulation treatment. ICH patients taking oral anticoagulants may not be sufficiently treated with platelet transfusion alone. This is because emergency treatment of anticoagulant-ICH requires rapid restoration of coagulation, which uses hemostatic factors such as PCC and recombinant factor VIIa, in addition to vitamin K, factor IX concentrates, and FFP. Emergency management of ICH is more difficult when the patient is also being treated with oral anticoagulants (39).

### Fresh Frozen Plasma (FFP) vs. Prothrombin Complex Concentrate (PCC) Transfusion

Few studies have drawn comparisons between the effects of FFP on patients with ICH and the use of PCC on these patients. A randomized trial by Steiner studied the two treatment options (FFP and PCC) for patients with ICH related to vitamin K antagonists (VKA-ICH). After administering FFP to 23 patients and PCC to 27 patients, it was discovered that the PCC was more effective regarding smaller hematoma expansion (27). The results showed that 8 of the patients administered FFP died, 5 from hematoma expansion within 48 h of symptom onset. The remaining 3 patients died from the initial ICH. In contrast, 5 of the patients administered PCC died, although it was reported that none of these deaths were from hematoma expansion. In this group, the first death occurred 5 days after the start of the PCC treatment and was due to cardiac arrest. Limitations of this study include a small sample size and early stoppage of the trial due to the significant differences in hematoma expansion between FFP and PCC. The study acknowledged the premature ending of the treatment as a reason for possible bias away from their null hypothesis (27). Further research involving a more extended study time is required to fully understand the differences between PCC and FFP on hematoma expansion. However, the data provided can still be used as a decent marker for the possible effects of PCC and FFP on hematoma expansion within the first 90 days of treatment.

In a retrospective study by Huttner et al., PCC was associated with a reduced incidence and hematoma expansion than FFP in patients who had ICH related to oral anticoagulant therapy (28). Hematoma growth was defined as a >33% increase in hemorrhage size compared to baseline measurements. The study emphasized that this result appeared to be related to the international normalized ratio (INR) reversal being more rapid in patients administered PCC. However, since this was a cumulative review, causality could not be determined. The study acknowledged the need for more randomized controlled trials to determine the best acute treatment to maintain INR reversal, given that higher INR levels were associated with hematoma expansion (28). From these studies, there appears to be an association between more effective maintenance of hematoma size and PCC use. When compared to PCC use, FFP treatment groups reported notably few patients who benefitted from treatment. Despite these results, each study had limitations that needed to be addressed, such as sample size, testing duration, and the nature of the study (retrospective). Although PCC may be a better option than FFP, more research is required with larger and more robust samples.

However, in situations involving warfarin, the results of using PCC vs. FFP become more ambiguous. Warfarin leads to hematoma expansion, as well as a higher incidence of ICH in 27–54% of cases, which partially explains an increase in mortality of up to 70% (87–90). The logical option is to rapidly reverse anticoagulation by substituting vitamin K for oral vitamin K antagonists to rapidly normalize coagulation (91).

### RBC Transfusion

**Figure 1:**
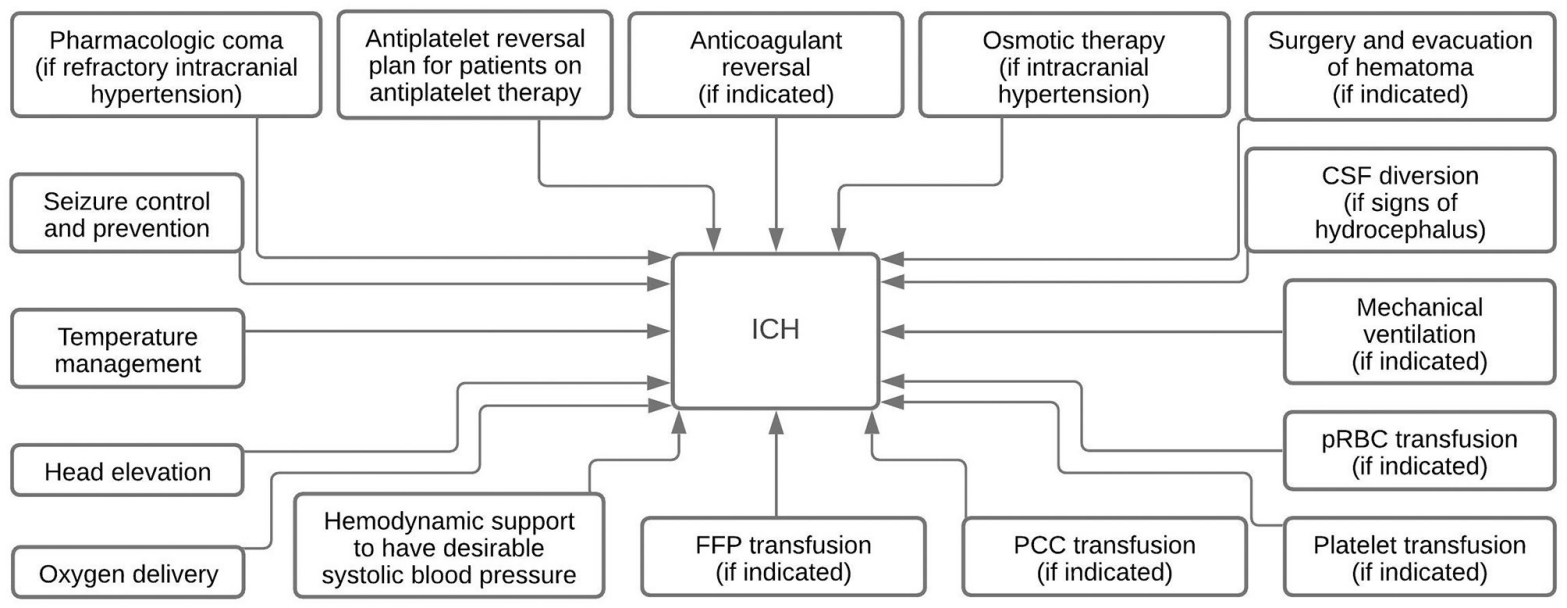
Summary diagram depicting various ICH treatment modalities.

Reviewing RBC transfusion therapy for ICH treatment reveals many conflicting results. Along with the general steps taken in the treatment of ICH, as shown in Figure 1, PRBC has also been used during the treatment of ICH. One study with positive results used PRBC transfusion to evaluate whether the therapy could mitigate ICH damage and anemia. Study outcomes showed improved survival rates at 30 days in 100 patients after receiving PRBC therapy (OR: 2.76, 95% CI: 1.45–5.26, *p* = 0.002) (33). Although this study expressed improved patient outcomes, it did not specify the change in hematoma expansion and volume and focused solely on anemia.

According to the WHO, anemia is defined as Hgb <12 g/dL for women and Hgb <13 g/dL for men. The volume of bleeding into the brain determines ICH outcome. Anemia is common in many critically ill patients. The presence of anemia in patients with ICH has been associated with larger ICH volumes. To date, two studies have evaluated anemia status in acute ICH, reporting that on admission, anemia was associated with larger hematoma volume and lower Hgb levels during a hospital stay, which was related to poorer outcomes. Anemia appears to be a predictor of unfavorable functional outcomes with independent factors beyond its association with a larger hematoma size. Recognizing anemia and the respective treatment may help influence the outcomes after ICH (92).

Critically ill patients commonly suffer from a lack of Hgb; 95% are anemic by day 3, and RBC transfusion is given to up to 50% of patients during their stay (93). Only three ICH studies are available that analyzed anemia and ICH in the context of oral anticoagulants. Diedler et al. in 2010 showed a direct relationship of nadir Hgb level during the hospital stay with functional outcomes in ICH (94). The study by Kumar et al. showed that anemia on admission was independently associated with greater hemorrhage volume increases, indicating through multivariate analysis that it may be possible for anemia to have effects on the outcomes that were not simply related to ICH size (72). Sheth et al. in 2011 reported improved survival rates 30 days after RBC transfusions during acute ICH treatment. Whether anemia is a marker for critical patients or whether it directly leads to increased hemorrhage volumes impacting outcomes remains to be proved (92). Mayer et al. and Anderson et al. in 2008 sought to answer this question by investigating the effect of anemia on outcomes in patients with minor-volume ICH (i.e., <30 cm^3^) (20, 95). This study showed that patients with anemia had larger baseline volumes and poorer neurological status.

Anemia had a positive but poorer association with larger hemorrhage volumes (AUC = 0.67), whereas the association with functional outcome was positive and more accurate for all patients with spontaneous ICH in another study by Kuramatsu in 2013 (AUC = 0.75; 95% CI: 0.70–0.80, *p* < 0.01). The study by Kuramatsu also showed worse outcomes in patients with minor-volume ICH with anemia. Despite similar characteristics, patients with anemia appeared to have a tendency for an increased rate of hemorrhage growth (*p* = 0.07). The relevance of anemia is even more striking in patients with minor-volume ICH. The only meaningful explanation for the observed outcome difference is anemia itself (92). Focusing on anemia upon admission facilitates in identifying high-risk patients with comorbidities and increased risk for hematoma expansion.

In a PRBC transfusion study by Chang et al., the unadjusted cumulative logit model reported that the odds of being discharged with an mRS of 5–6 were 9 times greater in transfused patients than in those who were not transfused (OR: 9.37, 95% CI: 2.84–30.88, *p* = 0.0002). When these data were adjusted for age, nadir Hgb, intubation time, and admitted ICH score, the data were no longer statistically significant, although there was still a positive association between PRBC transfusion and discharge with an mRS of 5–6 (OR: 4.01, 95% CI: 0.64–25.32, *p* = 0.1392) (31, 34). Overall, the results indicated that neither transfusion nor nadir Hgb could be used as independent predictors for in-hospital mortality (34). One consideration is the existence of confounding medical conditions that may have led to poorer outcomes in patients. Additionally, this study was limited by the small sample size and its retrospective nature. One factor that was not standardized was patient anemia levels upon admission; as other studies have noted, anemia at admission has been associated with larger hematoma volume, making it an essential consideration for standardization of this data (72).

Other studies indicate that RBC transfusion is associated with adverse effects in patients with ICH. In a study conducted by Ibrahim et al., patients who received PRBC treatment had an increased length of stay in health facilities and had similar morbidity and mortality rates as patients with ICH who did not receive the transfusion therapy. This study also implied that PRBC transfusion might cause worse outcomes, although it proposed that a larger sample size was needed to solidify this claim (30). Additionally, a retrospective study conducted by Roh et al. revealed that when using a large sample size of 597,046 patients with ICH, including 22,904 RBC transfusion patients, RBC transfusion was associated with increased odds of in-hospital mortality (adjusted OR: 1.22, 95% CI: 1.10–1.35, *p* < 0.001). Furthermore, after a sensitivity analysis, RBC transfusion correlated with worsened outcomes regardless of accounting for comorbidities and disease severity (adjusted OR: 1.43, 95% CI: 1.34–1.51, *p* < 0.001). Although the study incorporated a considerable sample size in its analysis from various databases, it was still a retrospective study and could not determine causality between RBC transfusion and outcomes in patients with ICH. The study acknowledged limitations, including a lack of granularity in the data and unavailable Hgb levels. This prevented the study from examining the direct connection of RBC transfusion and patient outcomes because other underlying diseases may have confounded the data for patients with ICH (29). Similar results were noted in a cohort study by Moman et al. that showed RBC transfusions resulted in longer hospital and intensive care unit stays for patients with ICH (32). Despite varied results, limited research exists on RBC transfusion and its effects on acute and delayed ICH injury, indicating that more in-depth research is required to further understand the effectiveness of RBC transfusion therapy after ICH.

### Tranexamic Acid

Studies have shown a link between the use of tranexamic acid and the mitigation of hematoma volume in patients with ICH. Tranexamic acid, a pharmaceutical agent, is a lysine-derived clotting promoter. In a randomized controlled trial of patients with non-traumatic ICH by Arumugam et al., tranexamic acid was linked to hematoma size maintenance after 24 h of administering the treatment compared to the placebo group (35). The control group's baseline median hematoma size was 14.53 cm^3^ compared to the post-24 h size of 17.59 cm^3^. The median difference of 3.07 cm^3^ was statistically significant (*p* = 0.001). On the other hand, the treatment group had a baseline value of 10.06 cm^3^ and a post-24 h size of 10.08 cm^3^, indicating no statistical difference (*p* = 0.313). Limitations of this study were that the patients received the treatment within 8 h of symptom onset. Although administering treatment at hour 8 can be helpful because hematoma expansion peaks from 4.5 to 12 h after symptom onset, the effectiveness of the treatment might not be the same for all time ranges. Some patients who might have had peak hematoma expansion at hour 12 may not have experienced the same effects of the treatment as patients who had a peak hematoma expansion at hour 5. Furthermore, the study was conducted with strict control of systolic blood pressure maintained at 140–160 mmHg, which led to the removal of two patients in the control group because of uncontrollable hypertension (35). To further elucidate the usefulness of tranexamic acid as a treatment option for acute and delayed ICH injury, studies that include a greater blood pressure range to assess the optimal time point of administration are needed.

Regarding the effects of blood pressure on tranexamic acid infusion for patients with ICH, a study by Gao et al. found that in spontaneous and traumatic ICH, patients who had moderate and severe hypertension (>160 mmHg) might be the more appropriate candidates for tranexamic acid treatment. Tranexamic acid was associated with a reduction in hematoma expansion (*p* = 0.002) and a decrease in hematoma volume (*p* = 0.03) compared to a placebo group. Patients with moderate to severe hypertension had an increased reduction in hematoma expansion rate (*p* = 0.02) and hematoma volume (*p* = 0.04) (36). Although this meta-analysis presented valid statistical interpretations of various trials, and although there appeared to be an association with tranexamic acid infusion and lower hematoma expansion, the study had several limitations. For example, the randomized controlled trial had significant gaps in sample sizes, and the results did not show whether tranexamic acid had different effects on hematoma expansion across genders. However, this meta-analysis has validity because it included data from 3,102 patients from multiple randomized controlled trials. In a systematic review by Hu et al., tranexamic acid led to an improvement in 90-day mortality (OR: 0.99, 95% CI: 0.84–1.18, *p* = 0.95). Additionally, reduction in hematoma expansion (OR: 0.54, 95% CI: 0.37–0.80, *p* = 0.002) and volume were noted (95% CI: −3.00 to −0.97, *p* = 0.0001) with a mean difference of −1.98 from the baseline value (37). The occurrence of single ischemic events and reported functional outcomes remained at statistically similar levels as the baseline; however, the use of tranexamic acid was associated with an increased risk of combined ischemic events such as myocardial infarction, deep vein thrombosis, ischemic stroke, transient ischemic attack, pulmonary embolism, or acute coronary syndrome (OR: 1.47, 95% CI: 1.07–2.01, *p* = 0.02) (37). Although the use of tranexamic acid may be associated with a reduced hematoma expansion rate and volume, these data indicate the potential risk of combined ischemic events associated with tranexamic acid treatment in patients with ICH. This finding limits the possible uses of tranexamic acid in patients with ICH and is a barrier for many patients who require an effective solution to hematoma expansion and volume and other factors such as edema. The study also reported that poor functional outcomes did not change, potentially because the degree of reduction in hematoma expansion and volume was not enough to do so or because the increase in combined ischemic risk counteracted these reductions (37). Furthermore, a limitation of this study is that it used data from only 14 studies. A larger sample size might be required to better understand the factors involved in using tranexamic acid in patients with ICH.

## General Considerations

Proper education and training are essential considerations regarding correctly transfusing patients, including adequate hospital blood product management and health quality improvement programs. Currently, our healthcare protocols would benefit from improved standards and regulations for transfusion therapy, given the inconsistencies in proposed research data. The lack of clinical transfusion guidelines for patients with ICH has led to unclear outcomes and an extensive knowledge gap in research. It would be helpful to incorporate a specific hospital-wide oversight program to provide an evidence-based approach for patients with ICH. Healthcare neuro-ICU professionals should evaluate specific patients with ICH who would potentially benefit from rigorous blood transfusion therapies. A multidisciplinary approach with contributions from critical care, vascular neurosurgery, vascular neurology, and rehabilitation medicine may help explain the efficacy of transfusion therapies (96). Adverse effects, such as hemolysis and iron overload, can be mitigated by creating standard protocols, for example, restricting the number of transfusions given to a patient and carefully transfusing only the amount necessary to achieve the clinical goal. A restrictive approach to blood transfusion may help to lower the overall cost of treatment. Financial considerations of blood transfusions are important because at least 50% of high-frequency transfusions are improperly administered to patients and, therefore, may place a financial strain on patients (97). A reduction in the frequency of blood transfusions may be possible if the effects of blood withdrawals, surgery, and other anemia-provoking procedures on the patient are considered. Protocols that properly screen for anemia and insufficient iron levels are associated with reduced blood transfusion frequency, length of stay, and overall cost (98).

It would be beneficial to provide alternative blood transfusion methods in patients when time is critical. The COVID-19 pandemic has increasingly strained health facilities, so much so that they may not be able to provide transfusion therapy to patients in a limited time frame. Methods to mitigate the spread of COVID-19, such as social distancing and stay-at-home orders, have contributed to the massive decrease in the amount of blood and platelet donations received. Furthermore, the influx of patients to hospitals worldwide has also contributed to low amounts of blood storage. We recommend efforts to research blood and platelet alternatives. Further research is also required to understand whether blood transfusion is safe during pandemic situations and how the viral spread may affect blood donation distribution. It would be helpful to consider optimizing the methods for properly storing and transporting packaged blood that emphasizes maintaining the contents' integrity.

Based on the mechanisms of ICH neurologic injury, several possible steps can be taken to alleviate ICH central and systemic symptoms. For example, the first step would be to reduce hematoma size through surgery. Another step would be to limit hematoma expansion. This can be possible by reversal of coagulopathy, hemostatic agents, or hypotensive therapy. Another step is modify molecular events, such as inflammation caused by Hgb degradation products, heme, and iron-mediated toxicity, or by quickening hematoma resolution (99).

## Conclusion

Currently, data are mixed regarding transfusions in patients with ICH. Various transfusion types appear to have different effects on hematoma size and expansion rates, as well as on edema and related secondary injuries. Some studies indicate that platelet transfusion is useful in promoting better patient outcomes; however, one study showed that it did not decrease in hematoma size. However, a mix of studies provides ambiguous conclusions, with some indicating better patient outcomes, some indicating worsened patient outcomes, and some showing no significant benefit from platelet transfusions. Furthermore, platelet transfusion in patients with ICH undergoing antiplatelet therapy has been controversial. RBC transfusion should theoretically provide better oxygen perfusion through tissues and allow for better patient outcomes, but this does not always appear to be the case. Many cases indicate that RBC transfusion may not be the best way to treat patients with ICH because it has not been shown to affect hematoma size and edema. However, patients with ICH who already have anemia could potentially use RBC transfusion, but these results appear to be controversial as well. It is vital to consider the reported adverse effects of transfusion therapies and critically evaluate their usefulness in every patient because simply increasing oxygen and Hgb levels may be detrimental. It may also be helpful to understand the effects of storage on transfusion products and how they relate to treating hematoma, as well as edema. Studies that observe and experiment with tranexamic acid, FFP, and PCC have had more promising results. Notably, tranexamic acid or PCC have been shown to control, or at least maintain, hematoma expansion rates more effectively than other transfusable compounds. However, these studies have limitations that need to be addressed to provide a complete understanding of when and how to use these therapies. Furthermore, it is important to consider the ischemic complications such as deep vein thrombosis that may be associated with tranexamic acid use. Such complications may counteract the benefits of this drug on patient outcomes. Anticoagulation reversal is also an important point to understand when treating patients with ICH with transfusion, as outlined in the discussion. Future research considerations include accounting for ICH severity, patient comorbidities, proper packaging of transfusable content, and RBC fragility. Further research is also required to fully understand the effects of each transfusion type on hematoma expansion rate and edema, as well as the associated secondary injuries.

## Author Contributions

SD: conceptualization and funding acquisition. SK and SD: methodology and analysis and writing—original draft preparation. SK, MA, AS, SA, MF, RA, and SD: writing—review and editing. All authors have read and agreed to the published version of the manuscript.

## Funding

Funding to support this work was provided by grants from the NIH (R21NS110008, R21NS103036, R21NS095166, and R56NS116076), Brain Aneurysm Foundation, DOD (AZ180127), and American Heart Association, and the Department of Anesthesiology (University of Florida College of Medicine, Gainesville, FL).

## Conflict of Interest

The authors declare that the research was conducted in the absence of any commercial or financial relationships that could be construed as a potential conflict of interest.

## Publisher's Note

All claims expressed in this article are solely those of the authors and do not necessarily represent those of their affiliated organizations, or those of the publisher, the editors and the reviewers. Any product that may be evaluated in this article, or claim that may be made by its manufacturer, is not guaranteed or endorsed by the publisher.
